# ALIGNING MENTAL HEALTH CARE FOR MARGINALIZED OLDER ADULTS: CLINICAL AND PROFESSIONAL DEVELOPMENT CONSIDERATIONS

**DOI:** 10.1093/geroni/igae098.0315

**Published:** 2024-12-31

**Authors:** Brenna Renn, Kristopher Kern

**Affiliations:** Chair; Discussant

## Abstract

As the older adult population grows and diversifies, the urgency mounts for clinicians and academics to provide inclusive, culturally responsive, and anti-racist services and training. Culturally responsive practice can improve care engagement and optimize patient outcomes. Attending to such issues across training ensures retention of students from marginalized backgrounds and prepares clinicians who reflect diversity of the aging population. This symposium convenes speakers from the Society of Clinical Geropsychology to discuss the challenges and opportunities in provider training and mental health service provision with a focus on inclusivity. Each presentation pays close attention to actionable steps. The first presenter outlines strategies to strengthen mentorship by fostering equity, diversity, and inclusivity. The second speaker presents qualitative findings regarding challenges, opportunities, and concrete strategies to improve culturally responsive and anti-racist care from a sample of geropsychologists and trainees across the U.S. The third presenter describes a national survey of geropsychology workforce diversification and presents inclusive strategies to support diverse learners at all stages of training. Finally, our Discussant synthesizes these findings in the broader context of training and clinical practice that is responsive to the needs of individuals from historically marginalized backgrounds and intersectional identities. The Discussant will comment on the pressing need to transform both how training is conducted and how services are delivered to ensure optimal outcomes. The insights gleaned from these three initiatives will lead to a rich discussion among the panel and audience on applications and future directions in geriatric mental health care with relevance across settings.

